# Development and characterization of chimeric antigen receptor macrophages for amyloid clearance

**DOI:** 10.3389/fimmu.2026.1783851

**Published:** 2026-03-06

**Authors:** Manasi Balachandran, James S. Foster, Trevor J. Hancock, Emily B. Martin, Joseph W. Jackson, Nicolas Angell, Jonathan S. Wall

**Affiliations:** 1 Department of Medicine, University of Tennessee Health Science Center, College of Medicine, Knoxville, TN, United States; 2 Attralus Inc., Naples, FL, United States

**Keywords:** amyloidosis, chimeric antigen receptor, macrophages, phagocytosis, polybasic peptides

## Abstract

Systemic amyloidosis is a protein folding disorder characterized by the extracellular deposition of protein fibrils in tissues and vital organs, leading to dysfunction and mortality. While there are monoclonal antibody-based therapies that promote cell-mediated amyloid clearance in various stages of clinical development, there are currently no treatment options which focus on reducing the tissue amyloid burden. Therefore, the urgent need for a transformative approach to facilitate amyloid clearance and restore organ function remains paramount. We demonstrate that a short, polybasic peptide (p5) can serve as a versatile recognition motif for chimeric antigen receptors in macrophages, enabling pan-amyloid binding and uptake. By comparing CH2- and CH3-spacer designs, quantifying glycan interactions, and establishing opsonization- and complement-dependent enhancement, we provide a blueprint for peptide-targeted CAR-M engineering beyond conventional scFv recognition. These findings broaden the repertoire of CAR targeting strategies and motivate translational studies of CAR-M for systemic amyloidosis, where established fibrils persist despite precursor-lowering therapies.

## Introduction

The group of progressive debilitating diseases characterized by the deposition of protein fibrils in the extracellular space of tissues and organs are collectively known as systemic amyloidosis (1). Amyloid deposits can disrupt tissue architecture and normal cellular function, leading to potentially life-threatening organ dysfunction. To date, approximately 20 heterogenous proteins have been identified as components of fibrils in patients with systemic amyloidosis (2). The most common types of disease arise from the deposition of variant or wild-type transthyretin (ATTRv and ATTRwt), monoclonal immunoglobulin light chains (AL), serum amyloid A (AA), or leukocyte chemotactic factor 2 (ALECT2). A small fraction of systemic amyloidosis are hereditary, caused by germline mutations in specific amyloidogenic proteins, but many systemic amyloidosis are acquired due to processes such as plasma-cell dyscrasias (e.g., AL) or chronic inflammation in which sustained overproduction of the acute-phase reactant, serum amyloid A, leads to its misfolding and deposition as insoluble amyloid fibrils in tissues and organs (e.g., AA) (2). Amyloid proteins adopt a predominantly β-pleated sheet structure when they are incorporated into insoluble fibrils (3) and can deposit in any organ or tissue, including the heart, kidneys, liver, spleen, nervous system, and gastrointestinal tract. The amyloid fibrils are resistant to proteolysis and the amyloid deposits are not generally recognized by the phagocytic cells of the innate immune system or the humoral immune system (4). Extensive amyloid deposition can often be observed with cardiac and renal amyloidosis representing the leading causes of mortality among affected patients (5).

Treatment options for systemic amyloidosis are generally limited to those patients with AL, ATTR or serum amyloid protein A (AA) amyloidosis and primarily aim to lower circulating precursor proteins preventing their incorporation into the amyloid fibril formation pathway (6). For the remaining types of systemic amyloid diseases, no treatments are available and transplantation of affected organs remains the principle option (7). In AL amyloidosis, therapy targets the monoclonal plasma cell population producing the amyloidogenic light chain, typically using protease inhibitors and immunochemotherapy; cyclophosphamide, bortezomib, dexamethasone and daratumumab (CyBorD) (8). In ATTR, two complimentary approaches are used: stabilization of the native tetramer using small molecules (9) and gene silencing to reduce hepatic transthyretin production (10); both approaches lower amyloidogenic monomeric levels and slow disease progression (11). Critically, no approved therapy is designed to remove established tissue amyloid, underscoring the need for innovative treatments that can selectively recognize and clear deposits to restore organ function.

Though adverse events and therapy resistance persist, recent advances in cellular immunotherapy, notably CAR-T cell therapies, have shown impressive results in treating hematological cancers (12). CAR-T therapies targeting B-cell maturation antigen (BCMA) for the treatment of multiple myeloma, may also show benefit AL amyloidosis (13). However, unlike tumors, systemic amyloid deposits are acellular and immunologically silent, limiting natural immune clearance and posing unique challenges for CAR-based therapies (14). To expand the therapeutic scope of CAR-based immunotherapies, researchers are exploring the use of macrophages, key innate immune cells known for their phagocytic capabilities and role in immune regulation. CAR-macrophages (CAR-M) are genetically engineered to express chimeric antigen receptors that recognize and respond to specific antigens (15). Unlike CAR-T cells, CAR-Ms leverage the natural tissue-infiltrating and phagocytic properties of monocytes/macrophages, potentially allowing for more effective clearance of pathological targets (15–17). This emerging class of immunotherapy holds promise beyond oncology, particularly for diseases like systemic amyloidosis where host immune responses are limited (4, 18–20).

We have previously developed a panel of pan-amyloid reactive peptides, including peptide p5, which is a synthetic polybasic peptide that has been shown to bind an electrostatic motif found in all amyloid fibrils (21). Peptide p5 exhibits selective binding to amyloid deposits, mediated by electrostatic interactions with highly ordered β-sheet–rich fibrils and the amyloid-associated hypersulfated heparan sulfate glycosaminoglycans. Prior *in vitro* and *in vivo* studies have demonstrated that p5 and the related polybasic peptides p5R and p5 + 14 bind mature amyloid fibrils, with no reactivity demonstrated toward native amyloid precursor proteins (22, 23). This specificity makes peptide p5 and its’ related homologs invaluable tools for developing diagnostic imaging agents and therapeutics that target all types of systemic amyloidosis (22–26). Herein, we describe two exemplar amyloid-reactive CAR constructs expressed in human THP-1 monocytes, designated as CARM-2 and CARM-5, utilizing the peptide p5 as the amyloid binding motif. Both CAR-M constructs exhibited robust binding to amyloid fibrils and human amyloid extracts and demonstrated significantly increased amyloid phagocytosis compared to native THP-1 cells, supporting the feasibility of developing CAR-M therapeutics for broad-spectrum amyloid clearance.

## Materials and methods

### Cell lines

HEK-293T/17 and THP-1 cell lines were procured from the American Type Culture Collection (ATCC). Cells were maintained in DMEM/F12 1:1 medium (Cytiva) supplemented with 10% FBS (Cytiva) and 1% PenStrep and 100 nM gentamycin (Gibco) unless specified otherwise.

### Plasmid design

**Figure 1:**
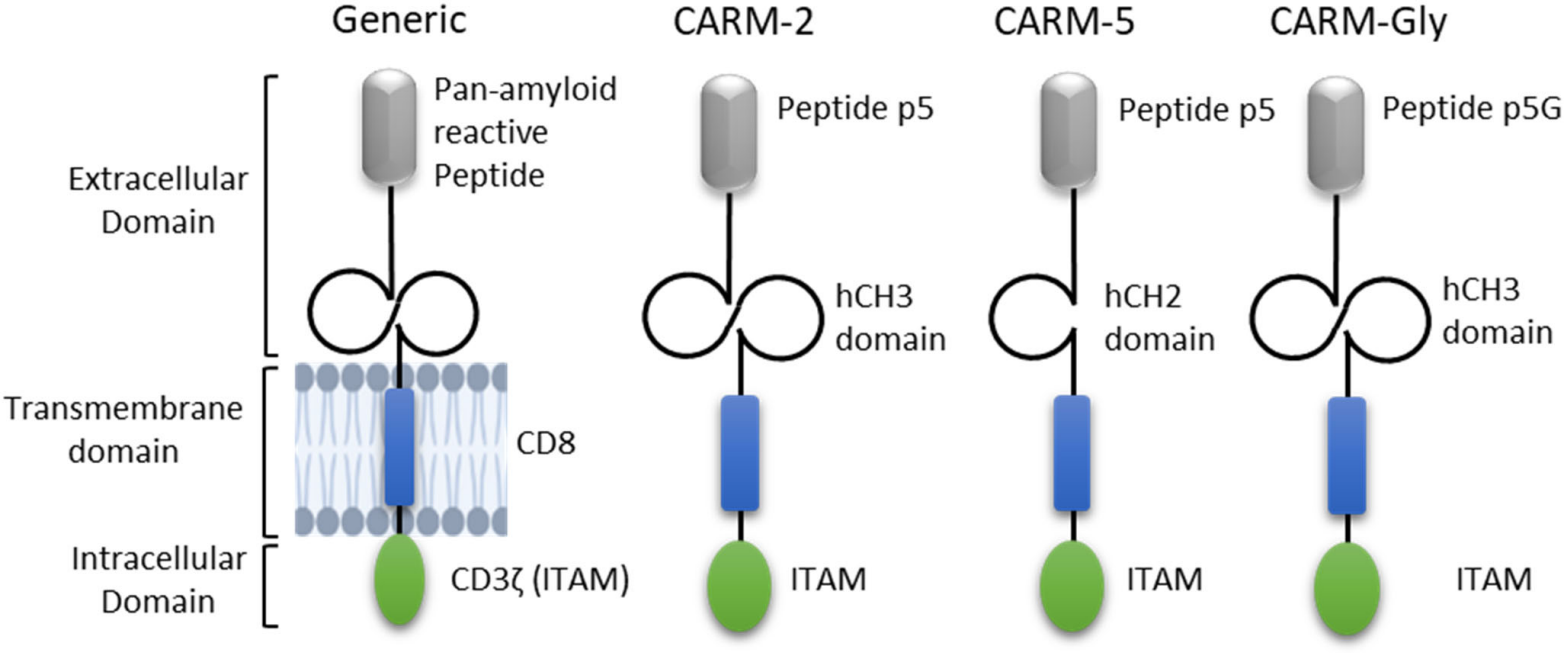
Schematic overview of the chimeric antigen receptor (CAR) variants. The extracellular domain comprises the pan-amyloid reactive peptide, p5 (grey) in CARM-2 and CARM-5, or p5G in CARM-Gly followed by either hCH3 or hCH2 domains (black). This is immediately followed by the CD8 transmembrane domain (blue) and a CD3ζ (green) as the signal transduction component with three ITAM domains.

The amyloid-reactive CAR construct contains approximately 345 amino acids comprising an extracellular domain terminating in the pan-amyloid reactive peptide p5 (GGGYSKAQKA QAKQAKQAQK AQKAQAKQAK Q) (**Figure 1**; **Table 1**). As a negative control, the uncharged peptide, p5G is used (GGGYSGAQGA QAGQAGQAQG AQGAQAGQAG Q), which does not bind amyloid. C-terminal to the peptide is a human immunoglobulin heavy chain domain CH3 in CARM-2 (QVSPSVGQPR EPQVYTLPPS RDELTKNQVS LTCLVKGFYP SDIAVEWESN GQPENNYKTT PPVLDSDGSF FLYSKLTVDK SRWQQGNVFS CSVMHEALHN HYTQKSLSLS PGKGGGGSGG GGSGGGGSGQ PREPQVYTLP PSRDELTKNQ VSLTCLVKGF YPSDIAVEWE SNGQPENNYK TTPPVLDSDG SFFLYSKLTV DKSRWQQGNV FSCSVMHEAL HNHYTQKSLS LSPGK) or CH2 in CARM-5 and CARM-Gly (QVSPSVPELL GGPSVFLFPP KPKDTLMISR TPEVTCVVVD VSHEDPEVKF NWYVDGVEVH NAKTKPREEQ YNSTYRVVSV LTVLHQDWLN GKEYKCKVSN KALPAPIEKT ISKAK) domain. The hCH2/hCH3 domains provide spacing and rigidity to the CAR structure. The constant domain is followed by a human CD-8 transmembrane domain (LSPGKTTTPA PRPPTPAPTI ASQPLSLRPE ACRPAAGGAV HTRGLDFACD IYIWAPLAGT CGVLLLSLVI TLYC) and finally an intracellular CD3ζ that consists of three immune receptor tyrosine-based activation motifs (ITAMs) to promote phagocytosis (RVKFSR SADAPAYQQG QNQLYNELNL GRREEYDVLD KRRGRDPEMG GKPQRRKNPQ EGLYNELQKD KMAEAYSEIG MKGERRRGKG HDGLYQGLST ATKDTYDALH MQALPPR).

**Table 1:** Primary sequence of the three CAR variants in this study.

Name	Sequence
CARM-2	MALPVTALLL PLALLLHAAR PSQFRVSPSV GGGYSKAQKA QAKQAKQAQK AQKAQAKQAK QVSPSVGQPR EPQVYTLPPS RDELTKNQVS LTCLVKGFYP SDIAVEWESN GQPENNYKTT PPVLDSDGSF FLYSKLTVDK SRWQQGNVFS CSVMHEALHN HYTQKSLSLS PGKGGGGSGG GGSGGGGSGQ PREPQVYTLP PSRDELTKNQ VSLTCLVKGF YPSDIAVEWE SNGQPENNYK TTPPVLDSDG SFFLYSKLTV DKSRWQQGNV FSCSVMHEAL HNHYTQKSLS LSPGKTTTPA PRPPTPAPTI ASQPLSLRPE ACRPAAGGAV HTRGLDFACD IYIWAPLAGT CGVLLLSLVI TLYCRVKFSR SADAPAYQQG QNQLYNELNL GRREEYDVLD KRRGRDPEMG GKPQRRKNPQ EGLYNELQKD KMAEAYSEIG MKGERRRGKG HDGLYQGLST ATKDTYDALH MQALPPR
CARM-5	MALPVTALLL PLALLLHAAR PSQFRVSPSV GGGYSKAQKA QAKQAKQAQK AQKAQAKQAK QVSPSVPELL GGPSVFLFPP KPKDTLMISR TPEVTCVVVD VSHEDPEVKF NWYVDGVEVH NAKTKPREEQ YNSTYRVVSV LTVLHQDWLN GKEYKCKVSN KALPAPIEKT ISKAKTTTPA PRPPTPAPTI ASQPLSLRPE ACRPAAGGAV HTRGLDFACD IYIWAPLAGT CGVLLLSLVI TLYCRVKFSR SADAPAYQQG QNQLYNELNL GRREEYDVLD KRRGRDPEMG GKPQRRKNPQ EGLYNELQKD KMAEAYSEIG MKGERRRGKG HDGLYQGLST ATKDTYDALH MQALPPR
CARM-Gly	MALPVTALLL PLALLLHAAR PSQFRVSPSV GGGYSGAQGA QAGQAGQAQG AQGAQAGQAG QVSPSVGQPR EPQVYTLPPS RDELTKNQVS LTCLVKGFYP SDIAVEWESN GQPENNYKTT PPVLDSDGSF FLYSKLTVDK SRWQQGNVFS CSVMHEALHN HYTQKSLSLS PGKGGGGSGG GGSGGGGSGQ PREPQVYTLP PSRDELTKNQ VSLTCLVKGF YPSDIAVEWE SNGQPENNYK TTPPVLDSDG SFFLYSKLTV DKSRWQQGNV FSCSVMHEAL HNHYTQKSLS LSPGKTTTPA PRPPTPAPTI ASQPLSLRPE ACRPAAGGAV HTRGLDFACD IYIWAPLAGT CGVLLLSLVI TLYCRVKFSR SADAPAYQQG QNQLYNELNL GRREEYDVLD KRRGRDPEMG GKPQRRKNPQ EGLYNELQKD KMAEAYSEIG MKGERRRGKG HDGLYQGLST ATKDTYDALH MQALPPR

### Lentiviral constructs, lentivirus production and concentration

The genes for CAR were synthesized and cloned into pLenti-C-mGFP-P2A-Puro Lentiviral Gene Expression Vector (Genscript). The plasmids were transfected into HEK-293T T/17 cells to package and generate lentiviral particles. Briefly, 2.5x10^7^ HEK-293 T/17 were plated in a T175 flask in DMEM/5% FBS and incubated at 37 °C in 5% CO_2_. Twenty-four hours after plating, plasmids pRSV/Rev (Addgene #12253), pMDLg/pRRE (Addgene #12251), pMD2.g (Addgene #12259) and pLenti-C-P2A-Puro (CAR) were mixed at a ratio of 1.25:1.25:6.25:6.25 together with polyethylenimine (PEI) (MW 25,000 – Polysciences, Inc) in DMEM (Cytiva) without FBS or antibiotics. DNA/PEI complexes were allowed to form for 20 minutes at room temperature and added to the cells for incubation at 37 °C in 5% CO_2_ for 4 hours. The transfection media was replaced with DMEM/5% FBS and the cultures incubated for 48 hours. The media containing the lentivirus was removed, centrifuged at 600 x g and filtered through a 0.45 μm pore-sized filter. Lenti viral supernatants were aliquoted and stored at -80 °C until use.

### Transduction of THP-1 cells with CAR lentivirus and single cell clone generation

Actively growing THP-1 cells (~1x10^6)^ were transduced with purified lentiviral supernatant (MOI ~5-10) mixed with 8 μg/ml polybrene (Millipore-Sigma) in complete DMEM/F12 medium. After 48 hours, cells were transferred into selection medium containing 1 μg/ml puromycin (Millipore-Sigma) for 5–7 days and the resistant cells were transferred into fresh media for expansion. THP-1 cells that were stably transduced with CAR and selected by puromycin were plated at a density of 1 cell/well in a 96-well plate by limiting cell dilution to generate single cell clones that were further expanded and characterized.

### Fibrils and amyloid extracts

Amyloid-like fibrils composed of recombinant λ6 WIL light chain variable domain (rVλ6Wil) were prepared in PBS with 0.05% sodium azide as previously described (27, 28). Briefly, rVλ6Wil protein was expressed in *E. coli* and purified from the periplasmic space. Following lyophilization, protein was resuspended in PBS and shaken at 37 °C for 48–72 hours to induce fibril formation. Fibril content was confirmed by Thioflavin T (ThT, Millipore Sigma) fluorescence analysis (excitation = 450 nm, emission = 490 nm). Human amyloid extracts were prepared from autopsy-derived tissues from patients with ATTRwt, ATTRv, ALλ (designated CLA) or ALκ (TAL) associated amyloidosis using the water flotation method, as previously described (28). For uptake studies, rVλ6WIL fibrils and human amyloid extracts suspended in DPBS (Cytiva) were labeled with pHrodo red fluorescent dye (Invitrogen) and washed as previously described (4). The use of human-derived materials was approved by the University of Tennessee Health Science Center, College of Medicine-Knoxville Institutional Review Board.

### Staining for CAR expression using microscopy

THP-1 and CAR-M cells (~7*10^5^/ml) were plated in 35 mm glass culture dishes (Cellvis). Phorbol-12-myristate-13-acetate (PMA, Millipore Sigma) was added at a final concentration of 50 ng/ml and the cells were allowed to differentiate for 24 hours. Thereafter, media containing PMA was removed and replaced with fresh media and the cells were allowed to ‘rest’ for 48 hours. Cells were washed with DPBS and fixed with 4% paraformaldehyde solution for 10 minutes at room temperature. Fixed cells were either stained immediately or stored at 4 °C until further use. For surface staining, cells were treated with 1 μg/ml goat anti-human IgG AlexaFluor-488 FcRγ fragment-specific antibody (Jackson Immuno Research, #109-545-190). Alternately, cells were stained with biotinylated anti-p5 antibody (in-house monoclonal antibody, clone 13-2) (29) followed by goat anti-mouse IgG antibody (H+L) AlexaFluor-594 (Jackson Immuno Research, #115-585-062). Non-transduced THP-1 cells served as a negative control. Following incubation for an hour, the cells were washed twice and stained with Hoechst nuclear stain (Life Technologies) for 10 minutes in the dark and were mounted using fluorescent mounting medium (Dako). Fluorescence images were acquired using a Keyence BZ-X800 fluorescence microscope (V 1.3.1) equipped with standard filter cubes. The blue channel was used for Hoechst nuclear stain (excitation ~360 nm, emission ~460 nm), the green channel for AlexaFluor-488 or CMFDA (excitation ~490 nm, emission ~510 nm), and the red channel for AlexaFluor-594 or pHrodo Red (excitation ~580 nm, emission ~600 nm). Images were collected using identical exposure settings within each experiment and processed uniformly across conditions.

### Confocal microscopy for CAR surface expression

THP-1 cells and CAR-M cells were plated in 35 mm glass culture dishes (Cellvis), differentiated with PMA, fixed and stained as described above. Confocal microscopy was performed at the Advanced Microscopy & Imaging Center (AMIC) at the University of Tennessee, Knoxville, using a Leica SP8 or Stellaris White Light Laser confocal microscope. Fluorescence signals were detected using hybrid (HyD) detectors, and images were acquired using a 63x objective lens by sequential scanning to prevent spectral crosstalk.

### Staining for CAR expression using flow cytometry

For surface staining, ~1x10^6^ THP-1 CAR-M cells were washed twice with DPBS (Cytiva) and blocked with 5% goat serum. Cells were stained with 1 μg/ml goat anti-human IgG AlexaFluor-488 (Fcγ fragment-specific antibody). Alternately, cells were stained with biotinylated anti-p5 antibody (in-house monoclonal antibody, clone 13-2) followed by AlexaFluor-488/594 goat anti-mouse IgG antibody (Jackson Immuno Research) as described previously. Untransduced THP-1 cells served as negative control. Following incubation for an hour, the cells were washed twice, resuspended in DPBS, and analyzed by flow cytometry using a 2-laser Cytek Northern Lights spectral flow cytometer (405 and 488 nm lasers) (Cytek Biosciences). For cytoplasmic staining, ~1x10^6^ THP-1 CAR-M cells were fixed with 4% paraformaldehyde solution followed by permeabilization with 0.2% Triton X-100. Cells were then blocked, stained as above, and analyzed by flow cytometry as previously described. Events were gated by Boolean analysis with doublet discrimination performed, and percent positive cells and mean fluorescent intensity (MFI) were calculated. Data were analyzed using FlowJo software version 10.8. (Becton, Dickinson & Company). Representative gating strategy is shown in **Supplementary Figure S1**.

### Cell Staining with CMFDA

Cells were transferred into a microcentrifuge tube and centrifuged at 200 x g for 5 minutes. The supernatant was aspirated, and the cell pellet washed twice with DPBS and resuspended in 1 ml of DPBS containing 1 μg of CellTracker™ green CMFDA (5-chloromethylfluorescein diacetate) dye (Invitrogen) and incubated for 15 minutes at 37 °C. The cells were washed twice with DPBS, resuspended in 2 ml RPMI, and incubated again for 15 minutes at 37 °C. The cells were washed twice as before, and the cell pellet was resuspended in 2 ml DPBS.

### Heparin binding assay

Twelve-well tissue culture-treated plates were pre-treated with 5 μg/ml poly-L-lysine (Millipore Sigma) overnight at 4 °C. The following day, plates were washed and coated with 100 μg/ml low molecular weight heparin (Enoxaparin Sodium Injection 100 mg/ml; Amphastar Pharmaceuticals Inc.) for 1 hour at room temperature. The plate was then blocked with 2% BSA-PBS for 1–2 hours at room temperature. A volume of 5x10^5^ CMFDA-stained CAR-M and non-transduced control THP-1 cells were added to the plate and incubated for 1 hour at 37 °C. The plate was washed with DPBS, and the amount of binding was determined by counting and quantifying the number of fluorescent particles captured on a fluorescence microscope (Keyence BZ X800 V 1.3.1) using ImageJ software (ver. 1.54p, National Institutes of Health, USA).

### *In Vitro* phagocytosis and complement activation assays

THP-1 and CAR-M cells (~1x10^6^/well) were plated in 24-well tissue culture-treated plates. Phorbol-12-myristate-13-acetate (PMA, Millipore Sigma) was added at a final concentration of 50 ng/ml and the cells were allowed to differentiate for 24 hours. After 24 hours, media containing PMA was removed, replaced with fresh media, and the cells were allowed to ‘rest’ for 48 hours. For the phagocytosis assay, the cells were washed with DPBS and 1 ml of RPMI media was added to each well. pHrodo red-labeled recombinant Wil fibrils (rVλ6WIL) and human amyloid extracts (ALκ, ALλ, ATTRwt and ATTRv) were added at a final concentration of 20 μg/ml to each well, and the plate was incubated at 37 °C for 1 hour to enable phagocytosis. Fluorescence microscopy (Keyence BZ X800 V 1.3.1) was used to quantify the amount of phagocytosis by monitoring the increased fluorescence emission of the pHrodo red as the dye enters the acidified phagolysosome. Phagocytosis was quantified by image segmentation of the fluorescence emission using Image Pro Premier software (Image Pro Premier V 9.0), with pHrodo Red–positive regions identified by uniform intensity thresholding. Fluorescence signal was normalized to cell number per field, determined by nuclear staining.

To determine the effect of opsonization and complement activation on phagocytosis of amyloid, experiments were performed in the presence of the amyloid-reactive antibody (10 nM zamubafusp alfa; AT-02) (23, 24, 27), a hIgG1 control antibody (10 nM), together with the CAR-M test article, in media supplemented with 20% citrate-treated human plasma as a source of complement factors.

### Statistical analyses

Unless otherwise indicated, data are presented as mean ± standard deviation derived from at least four technical replicates per experiment. Individual experiments were repeated at least three times and representative data have been shown wherever applicable. Correlation analyses were performed by determining the Pearson r using a two-tailed equation with 95% confidence interval. Comparison of phagocytosis data was performed using an unpaired Student t-test with α = 0.05 (when the data were normally distributed), or non-parametric equivalent. All analyses were performed using GraphPad Prism (Version 10.4.2).

### Ethics statement

All patient-derived samples were used in accordance with an Institutional Review Board-approved application.

## Results

### Expression of CAR in THP-1 cells

**Figure 2:**
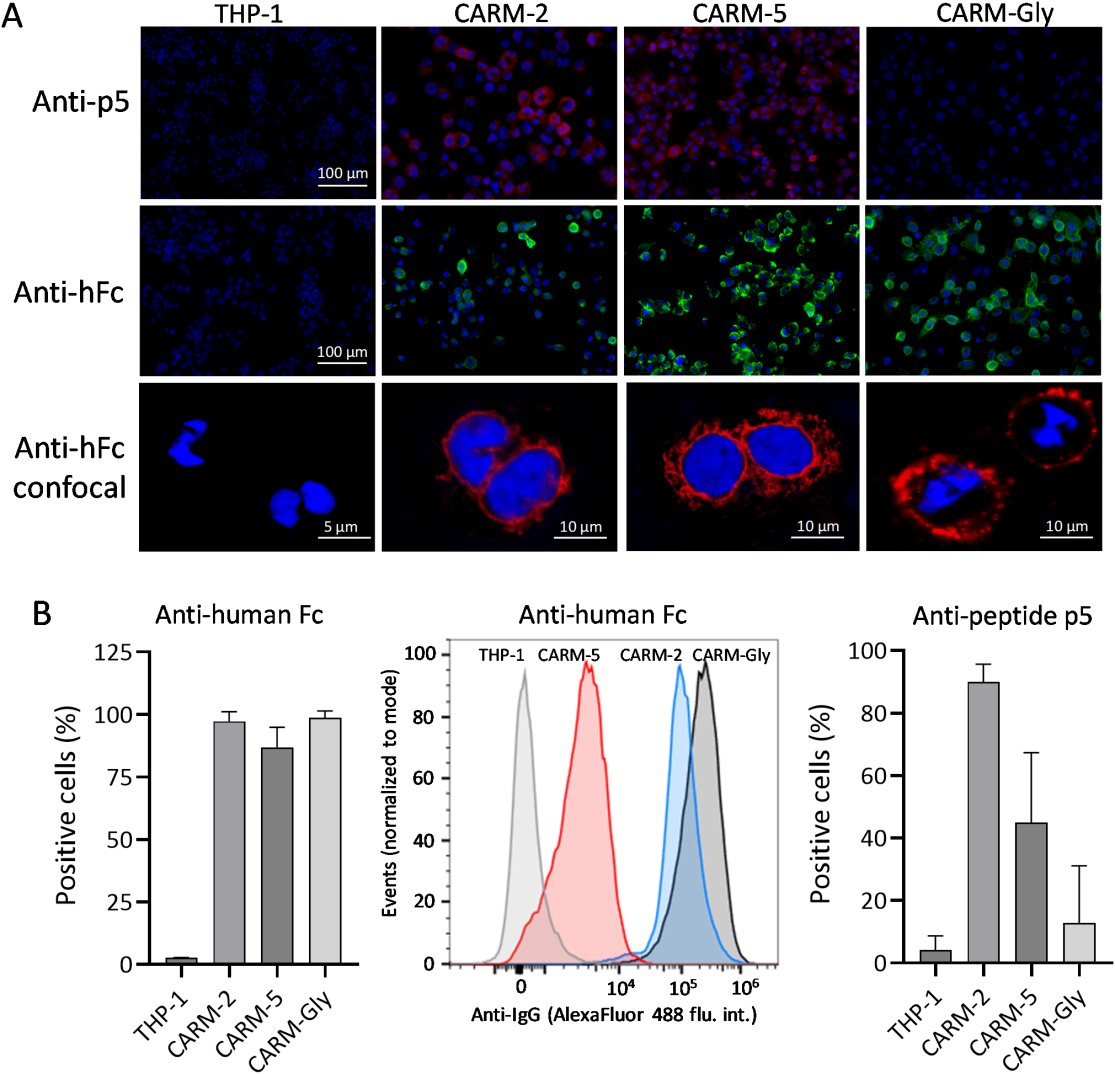
Surface expression and quantification of CAR in engineered THP-1 cells and untransduced control cells. **(A)** Immunostaining of CAR using anti-p5 antibody (top row, red) and anti-human Fc (middle row, green) visualized by fluorescence microscopy and confocal microscopy using anti-human Fc (bottom row, red) Cell nuclei are stained blue. **(B)** Percentage of positively stained cells evaluated by flow cytometry following incubation with either an anti-human Fc antibody (left), histogram (middle), or an anti-p5 antibody (right). Untransduced THP-1 cells served as a negative control.

The expression of CARs in the plasma membrane with appropriate orientation was assessed by immunostaining using intact (non-permeabilized) cells. Both the CARM-2 and CARM-5 showed positive immunostaining in the presence of an AlexaFluor-594-conjugated anti-p5 peptide monoclonal antibody whereas the non-transduced THP-1 and CARM-Gly showed no staining (**Figure 2A**).

The presence of the hCH2/CH3 domain (both components of the IgG Fc-domain) on the outer surface of the cells was visualized using an AlexaFluor-488-conjugated anti-human Fc antibody. In this case, all three CAR-M lines were shown to express the CAR construct on the cell surface with no staining of THP-1 cell surfaces (**Figure 2A**). Further confirmation of the presence of the CAR construct on the surface of the CAR-M lines was provided by confocal microscopy using an AlexaFluor-594-conjugated anti-human Fc reagent (**Figure 2A**).

Surface expression of the CAR was evaluated by flow cytometry using antibodies directed against the human Fc domain or the p5 peptide (**Figure 2B**). Staining with an anti-human Fc antibody demonstrated that the majority of cells in each CAR-M population expressed detectable levels of surface Fc, with minimal signal observed in untransduced THP-1 cells (**Figures 2B**, left). While Fc positivity was high across all CAR-M variants, the degree of separation from THP-1 controls and the distribution of signal intensity varied between CARM-2, CARM-5 and CARM-Gly.

In contrast, staining with an anti-p5 antibody revealed marked differences in peptide detectability between individual CAR variants (**Figures 2B**, right). CARM-2 cells exhibited a high proportion of p5-positive cells, whereas CARM-5 showed a reduced fraction of p5-positive cells. As expected, CARM-Gly with the glycine version of the peptide had no significant increase in reactivity with the anti-p5 peptide monoclonal antibody over untransduced THP-1 cells.

These findings suggest that while CAR surface expression is broadly maintained across constructs, spacer composition potentially influences peptide accessibility or stability at the cell surface.

### CAR-expressing THP-1 cells exhibit peptide-mediated heparin binding

**Figure 3:**
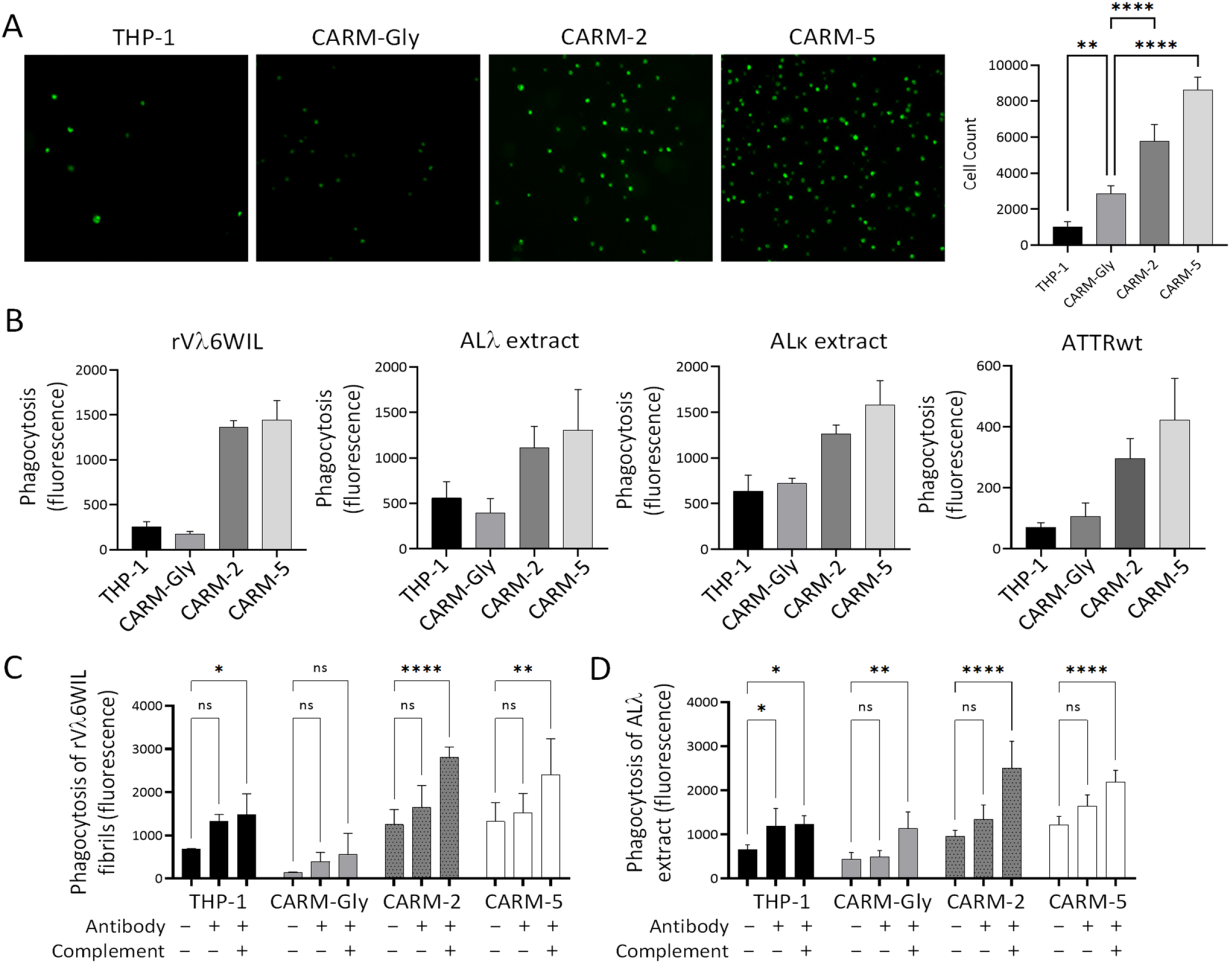
Functional assays to demonstrate CAR bioactivity *in vitro*. **(A)** Heparin-binding of CMFDA-labeled CARM-2, CARM-5, CARM-Gly and THP-1 control cells to heparin coated plates. **(B)** Phagocytosis of rVλ6WIL fibrils, human AL and ATTR amyloid extracts by PMA-activated CARM-2,5,Gly and THP-1 cells after 1 hour incubation. **(C, D)** Effect of opsonin (antibody zamubafusp alfa) and complement (20% citrate plasma) on phagocytosis of rVλ6WIIL fibrils and human AL amyloid extract. Data are representative of an independent experiment; values are presented as mean ± standard deviation of four technical replicates. Data were analyzed using a 2-way ANOVA with α = 0.05. *p < 0.05; **p < 0.01; ****p < 0.0001.

Peptide p5 binds amyloid fibrils and highly sulfated heparan sulfate species; heparin, although distinct from heparan sulfate, is commonly used as an experimental surrogate due to its high degree of sulfation. The bioactivity of peptide-CAR on the plasma membrane of cells was, therefore, assessed using a heparin binding assay. The interaction of CMFDA-labeled CAR-M with heparin coated microplate wells was evaluated by fluorescence microscopy. A higher density of CMFDA-labeled CARM-5 relative to CARM-2 was observed visually, with only sparse cell binding observed for THP-1 and CARM-Gly cells (**Figure 3A**). Following fluorescence image quantitation, relative to CARM-Gly cells both CARM-2 and CARM-5 showed significantly increased heparin-coated plate binding (*p* < 0.0001) (**Figure 3A**) with CARM-5 cells showing ~30% greater binding to the heparin-coated plate as compared to the CARM-2 cells.

### Amyloid-reactive CAR-M bioactivity

*Ex vivo* phagocytosis assays were performed using synthetic rVλ6WIL fibrils and patient-derived AL or ATTR amyloid extracts labeled with pHrodo red. THP-1 cells and CAR-transduced THP-1 cells were stimulated to M0 macrophage-like cells using PMA and incubated with pHrodo red-labeled rVλ6WIL fibrils or amyloid extracts. After 1 hour of incubation there was a significant >5-fold increase in the phagocytosis of rVλ6WIL fibrils by CARM-2 and -5 relative to the control CARM-Gly cells (**Figure 3B**). A smaller, two to three-fold increase in phagocytosis was observed when patient-derived ALλ, ALκ and ATTRwt amyloid extracts were used as the substrate (**Figure 3B**).

We next assessed the impact of an amyloid-reactive monoclonal antibody (zamubafusp alfa) or human plasma (20%), as a source of complement factors, on CAR-M-mediated phagocytosis with pHrodo red-labeled synthetic rVλ6WIL fibrils or a human ALλ amyloid extract. Addition of an amyloid-binding antibody, zamubafusp alfa, alone resulted in a slight, but typically not significant increase in phagocytosis of either substrate (**Figures 3C, D**). However, addition of zamubafusp alfa in the presence of 20% human plasma resulted in significant increases in the phagocytosis of both synthetic fibrils (**Figure 3C**) and ALλ amyloid extract (**Figure 3D**) by CARM-2 and CARM-5 cells. Significant increases in the phagocytosis of rVλ6WIL fibrils and ALλ amyloid extract by THP-1 cells was also observed following addition of the antibody and human plasma (**Figures 3C, D**).

### Correlation of phagocytic activity with CAR surface expression

**Figure 4:**
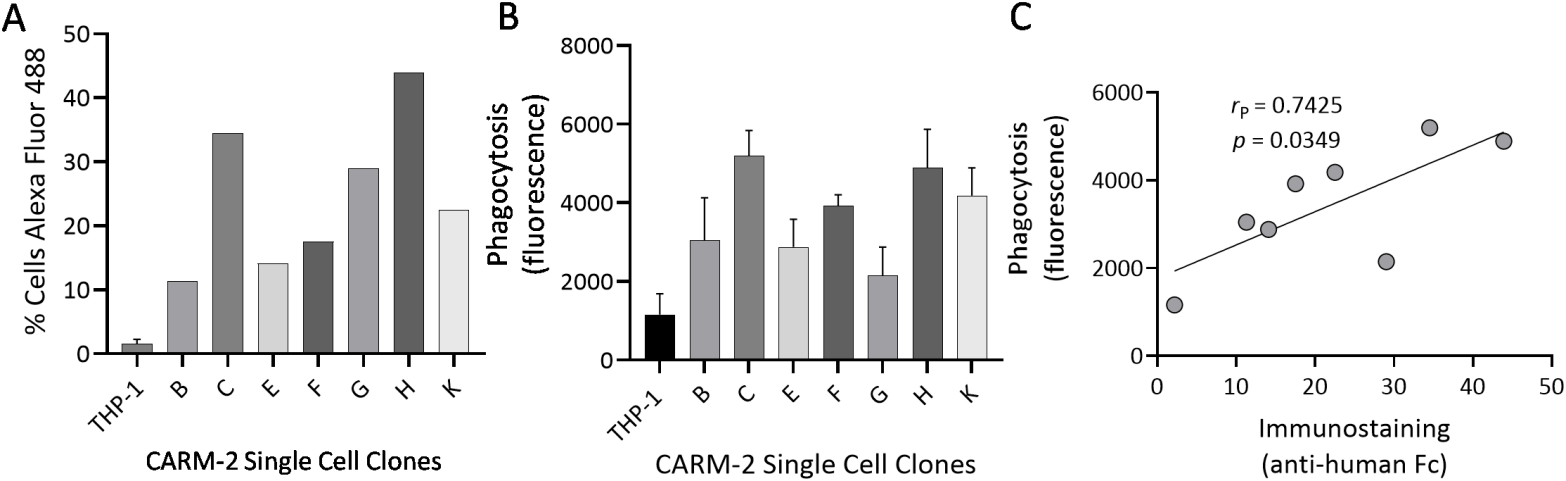
Surface expression of CAR in CARM-2 correlates with phagocytic activity. **(A)** Surface expression of CAR in CARM-2 single-cell clones (designated B, C, E, F, G, H, K) and untransduced control THP-1cells stained with anti-human Fc. **(B)** Phagocytic activity of single cell clones quantified by uptake of pHrodo red-labeled rVλ6WIL fibrils. **(C)** Correlation between surface expression and phagocytosis (Pearson *r* = 0.76). Additional characterization of CAR expression across single-cell clones is shown in **Supplementary Figures S2**, **S3**.

During subcloning of the CARM-2 and -5 pools to generate single-cell clones, we took the opportunity to assess whether cell surface expression of amyloid-reactive CAR correlated with phagocytic activity (**Figure 4** and). This was only possible for the CARM-2 pool, as the CARM-5 single cell clones consistently demonstrated >90% of cells expressing surface CAR (**Supplementary Figures S2**, **S3**).

The number of surface-CAR expressing cells in the single cell CARM-2 clonal population was assessed by flow cytometry following staining with AlexFluor-488-conjugated anti-human Fc antibodies. Expression varied (in absolute cell numbers without controlling for background staining seen in the THP-1 cells) from ~35% (clone E) to ~65% (clones C and H) (**Figure 4A**). The phagocytosis of pHrodo red-labeled rVλ6WIL fibrils similarly showed variability between the seven clonal populations (**Figure 4B**). The linear correlation between the number of cells expressing CAR and bioactivity was strong (Pearson *r* = 0.7425) and significant (*p* = 0.0349) (**Figure 4C**). The major outlier was clone G which exhibited relatively high cellular expression of CAR but the lowest phagocytosis values.

Additionally, CAR surface expression across individual CARM-2 and CARM-5 single cell clones demonstrated heterogeneity not only in the percentage of CAR-positive cells but also in the level of CAR expression per cell, as reflected by differences in AlexaFluor-488 mean fluorescence intensity (MFI) (**Supplementary Figures S2**, **S3**). Based on the combined assessment of CAR surface expression and phagocytic activity, clone CARM-2H and CARM-5F (unless otherwise indicated) were selected for subsequent experiments, as they exhibited robust CAR expression and reproducible amyloid uptake.

## Discussion

Amyloidosis comprises a heterogeneous group of disorders characterized by the deposition of amyloid fibrils in tissues and organs, leading to progressive dysfunction and a spectrum of morbidities. Despite advances in clinical management, currently approved therapies for amyloidosis, including precursor-suppressing agents, primarily slow disease progression but show limited efficacy in clearing pre-existing fibrils. The persistent presence of any amyloid burden continues to drive organ dysfunction, highlighting the need for therapies that actively promote amyloid removal. Recent strategies have focused on engaging the innate immune system, particularly macrophage-mediated phagocytosis, using monoclonal antibodies designed to engage macrophages that can clear amyloid. In this context, engineered chimeric antigen receptor macrophages (CAR-M) offer a promising cell-based immunotherapy platform to enhance targeted recognition and clearance of amyloid deposits, potentially enabling true disease modification. The pan amyloid-reactive peptide, p5, binds amyloid in a conformation-dependent manner preferentially binding β-sheet-rich amyloid fibrils and the amyloid-associated hypersulfated heparan sulfate glycosaminoglycans, with no observable binding to native monomeric amyloid precursor proteins. The studies described herein represent an initial demonstration that functional CAR-Ms targeting diverse amyloid types can be constructed based on the target recognition provided by the pan-amyloid binding peptide p5. This was demonstrated both through peptide-associated heparin binding (**Figure 3A**) and as enhanced amyloid uptake (**Figures 3B, C**).

Effective translation of CAR-M therapy into a viable treatment for systemic amyloidosis will require careful consideration of several key factors including structure and design considerations that support clinical safety and efficacy. Instead of pursuing the traditional scFv route as the target-recognition motif, the pan-amyloid peptide p5 was directly incorporated onto the human immunoglobulin CH2/CH3 (IgG1) domains. Both domains are components of the immunoglobulin Fc region and play distinct structural and functional roles. The CH2 domain is typically glycosylated at Asn297 and mediates key effector functions by interacting with Fcγ receptors and C1q, enabling antibody-dependent cytotoxicity and complement activation (30, 31). In contrast, the CH3 domain can form a stable homodimer that provides structural integrity to the Fc (32). These domains combine the potential for immune signaling and structural stability. CARM-2 and CARM-5 were selected to represent two structurally distinct Fc-derived spacer architectures for comparative evaluation rather than exhaustive spacer optimization.

The CAR surface expression analysis, using the anti-peptide antibody, suggests that the peptide moiety in the CARM-5 construct is less abundant on the surface expressed CAR relative to CARM-2 cells (**Figure 2B**). This difference between the two CAR constructs may reflect enhanced susceptibility of the CARM-5 peptide to extracellular proteolytic processing or conformational exposure that renders it more accessible to cell-surface or secreted proteases, or less accessible to the anti-peptide antibody. Conversely, the CARM-2 construct may confer greater structural protection, potentially due to differences in the plasma membrane presentation. Despite this apparent difference in the percent of cells expressing peptide p5 in CARM-2 and CARM-5 transduced cells, the heparin binding and phagocytic activity remains unaffected; indeed, the CARM-5 expressing cells exhibited greater activity (although not statistically significant for phagocytosis, but p=0.0001 for heparin binding) in both assays relative to CARM-2 (**Figures 3A, B**). This counterintuitive observation suggests that the presentation of the CAR in the plasma membrane in CARM-5 results in higher intrinsic bioactivity possibly indicating that the hIg CH2 spacer is more efficient in this context. In these studies, the heparin binding assay is used as a surrogate for the interaction with hypersulfated domains in amyloid-associated heparan sulfate glycans. We have previously demonstrated that peptide p5 does not bind heparan sulfate proteoglycans found in healthy organs and tissues. We have hypothesized that the specific binding of peptide p5, and its homologs, p5R and p5 + 14, is dependent on an interaction with a linear array of negative charges, such an organized highly negative surface charge density is found in amyloid fibrils and heparin (as a surrogate for the heparan sulfate associated with amyloid) (33). The human IgG-Fc domain can weakly bind heparin through electrostatic interactions, but this is not a canonical or high-affinity binding event like the interactions with FcγR, C1q, or FcRn (34, 35). This likely accounts for the higher binding of CARM-Gly to heparin-coated plates compared to untransduced THP-1 control (**Figure 2A**).

One advantageous feature of CAR-Ms relative to other cell-based therapies is the inherent plasticity and versatility within the complex pathological microenvironment. Macrophages possess the ability to adapt their phenotype and function based on signals from their surroundings, which includes interactions with soluble factors, stromal cells and the extracellular matrix. These interactions can potentially enhance the efficacy of CAR-Ms in targeting and phagocytosis of the target. In these preliminary studies, we have shown that the addition of human plasma as a source of complement and amyloid-opsonizing antibody can significantly enhance the phagocytosis of AL amyloid fibrils and human AL amyloid extracts (**Figures 3C, D**). These data suggest that the potential therapeutic activity of amyloid-reactive CAR-M could be enhanced by the presence of a depleter (amyloid-opsonizing antibody) and may be a useful adjunct to the use of depleters. Amyloid binding antibodies are under development (36, 37), and they have the ability, through complement fixation, to generate a pro-inflammatory microenvironment in systemic amyloid deposits that could inspire targeting of CAR-M to tissue amyloid. Systemic amyloid deposits are generally considered immunologically inert or shielded (38) and are not, with rarely documented exceptions, recognized by the innate or humoral immune system, which may be problematic with respect to CAR-M targeting.

The development of a clinically viable pan-amyloid reactive CAR-M for clearing systemic amyloid may provide a valuable tool for treatment of patients, particularly those for whom disease modifying therapies are unavailable. This path will require further studies to optimize the structure of the CAR, ensure safety, optimize activity by exploring the use of co-stimulatory domains and evaluate off-target effects *in vivo*. Demonstrating therapeutic efficacy in appropriate animal models is critical as is the importance of combining therapy with amyloid-reactive antibodies. Delivery of CAR-M to tissue could be explored particularly with respect to the rare types of localized amyloidosis where focused injection may be a viable early approach and clinical testbed.

In conclusion, chimeric antigen receptors that utilize pan-amyloid reactive peptides as the target recognition moiety are feasible and potentially clinically relevant. CAR-Ms represent a promising new avenue for the treatment of amyloidosis, perhaps made more attractive by the potential afforded by amyloid depleters that are in development. By enhancing the phagocytic capabilities of macrophages through CAR engineering, CAR-Ms hold the potential to enable tissue amyloid clearance. If successful, CAR-Ms may provide a transformative approach to improve the outcomes and quality of life for individuals affected by amyloidosis.

## Data Availability

All data and relevant figures pertaining to this study have been included in the main manuscript and/or in the **Supplementary Material**.
