# Supplementary material for: Development and characterization of chimeric antigen receptor macrophages for amyloid clearance
Source: Front Immunol. 2026 Mar 6;17:1783851. doi: 10.3389/fimmu.2026.1783851 (PMC13002434; doi:10.3389/fimmu.2026.1783851)
Supplement: Supplementary file 1 [file Presentation1.pptx]

## Slide 1
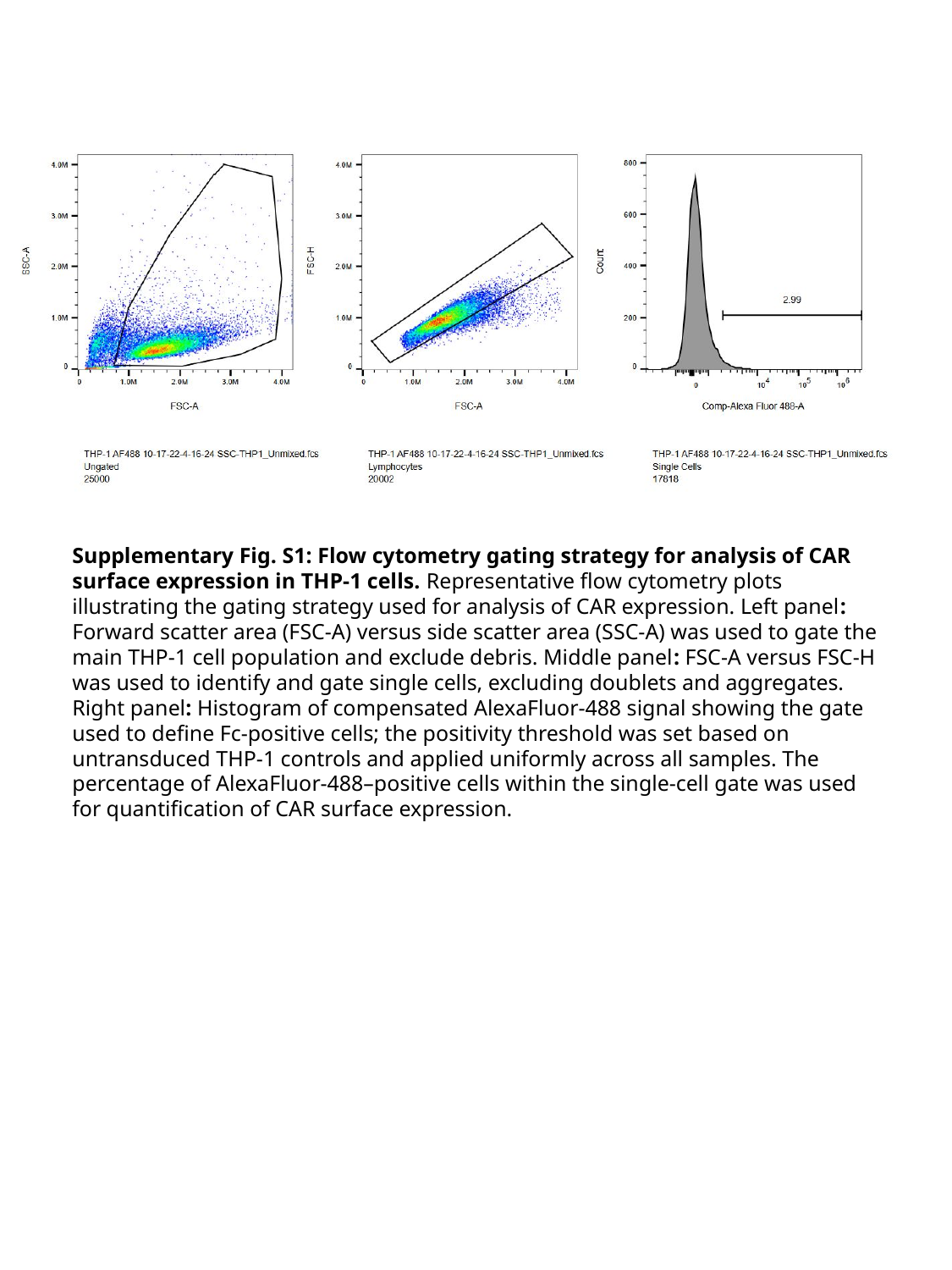

Supplementary Fig. S1: Flow cytometry gating strategy for analysis of CAR surface expression in THP-1 cells. Representative flow cytometry plots illustrating the gating strategy used for analysis of CAR expression. Left panel: Forward scatter area (FSC-A) versus side scatter area (SSC-A) was used to gate the main THP-1 cell population and exclude debris. Middle panel: FSC-A versus FSC-H was used to identify and gate single cells, excluding doublets and aggregates. Right panel: Histogram of compensated AlexaFluor-488 signal showing the gate used to define Fc-positive cells; the positivity threshold was set based on untransduced THP-1 controls and applied uniformly across all samples. The percentage of AlexaFluor-488–positive cells within the single-cell gate was used for quantification of CAR surface expression.

## Slide 2
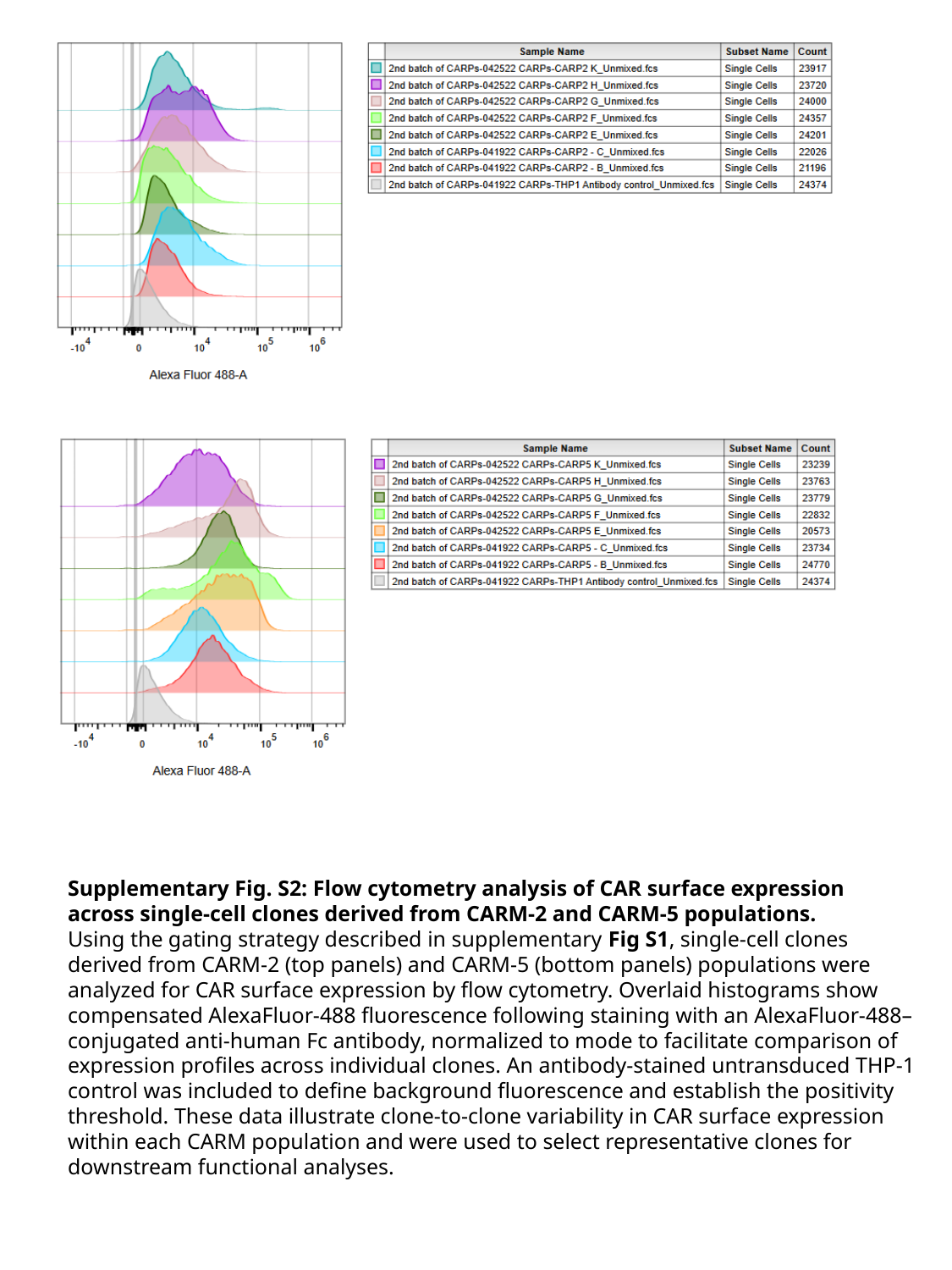

Supplementary Fig. S2: Flow cytometry analysis of CAR surface expression across single-cell clones derived from CARM-2 and CARM-5 populations.
Using the gating strategy described in supplementary Fig S1, single-cell clones derived from CARM-2 (top panels) and CARM-5 (bottom panels) populations were analyzed for CAR surface expression by flow cytometry. Overlaid histograms show compensated AlexaFluor-488 fluorescence following staining with an AlexaFluor-488–conjugated anti-human Fc antibody, normalized to mode to facilitate comparison of expression profiles across individual clones. An antibody-stained untransduced THP-1 control was included to define background fluorescence and establish the positivity threshold. These data illustrate clone-to-clone variability in CAR surface expression within each CARM population and were used to select representative clones for downstream functional analyses.

## Slide 3
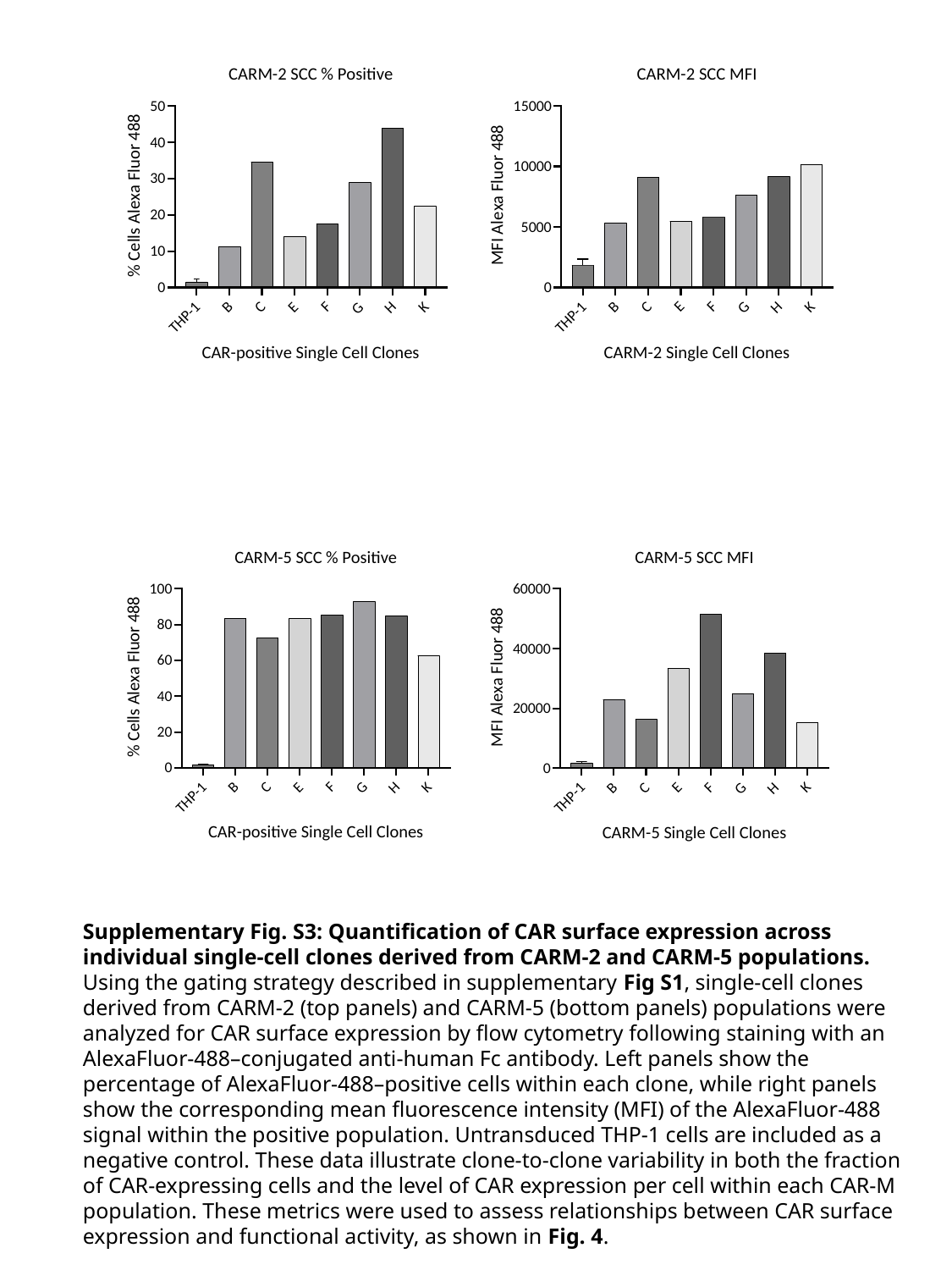

Supplementary Fig. S3: Quantification of CAR surface expression across individual single-cell clones derived from CARM-2 and CARM-5 populations.
Using the gating strategy described in supplementary Fig S1, single-cell clones derived from CARM-2 (top panels) and CARM-5 (bottom panels) populations were analyzed for CAR surface expression by flow cytometry following staining with an AlexaFluor-488–conjugated anti-human Fc antibody. Left panels show the percentage of AlexaFluor-488–positive cells within each clone, while right panels show the corresponding mean fluorescence intensity (MFI) of the AlexaFluor-488 signal within the positive population. Untransduced THP-1 cells are included as a negative control. These data illustrate clone-to-clone variability in both the fraction of CAR-expressing cells and the level of CAR expression per cell within each CAR-M population. These metrics were used to assess relationships between CAR surface expression and functional activity, as shown in Fig. 4.
